# Can aggressive postoperative non-narcotic therapy replace narcotics in patients undergoing laparoscopic hysterectomies?

**DOI:** 10.1186/1753-6561-9-S1-A5

**Published:** 2015-01-14

**Authors:** G Singh, SK Bates

**Affiliations:** 1 Royal College of Surgeons in Ireland, 123 St. Stephen’s Green, Dublin 2. Ireland; 2 Bates McMaster University, Hamilton, Ontario, Canada

## Background

Pain, although invariably present after surgical procedures, is not always well controlled. Medications from different analgesic groups are often used to control post-operative pain. Recently, attention has turned towards the optimization of non-opioid analgesics including NSAIDS and acetaminophen. After gynaecologic laparoscopy up to 80% of patients may require opioid analgesia. However, opioids can have adverse effects including nausea, vomiting, sedation, and respiratory depression. Thus the prudent surgeon attempts to use a narcotic-sparing approach to post-operative analgesia. Theoretically, aggressive non-narcotic analgesic administration will result in less narcotic use. Fortunately, both NSAIDs and acetaminophen are very effective in the control of moderate to severe pain and have few side effects. Our research question then is: “What is the post-operative narcotic use amongst women undergoing laparoscopic hysterectomy who receive aggressive non-narcotic therapy?”

## Methods

The subjects of interest were undergoing laparoscopic hysterectomy in a Canadian community hospital. Data from one calendar year was reviewed. For all patients the same routine pre-printed orders were used by the nursing staff. The order set included non-prn (non-discretionary) post-operative non-narcotic analgesics (ketorolac and acetaminophen). Narcotics were administered by the nursing staff on a prn basis for non-response/breakthrough pain after administration of the non-narcotic analgesics. Two databases, Meditech® and OR Manager® were used to extract information. Medication administration was determined from the Meditech® “Medications” module. Only ward administration of narcotics was included. All narcotics were converted to IV-morphine equivalents using Canadian Pharmacist Association (2008) morphine-centric equi-analgesic conversions. The data was tabulated and analyzed using Microsoft Excel.

## Results

Two hundred sixteen women underwent laparoscopic hysterectomy in the year ending July 30 2013. Meperedine, morphine, codeine, tramadol, and oxycodone were the narcotics administered. Overall, only 29% of the patients received narcotics. The mean narcotic dose in those patients who received narcotics was 4.1 morphine-equivalent mgs IV. Of those who received post-op narcotics 22% did so between hours 0 and 6 and 23% between hours 6 and 12. When between-surgeon comparison was performed there was marked variation in narcotic consumption by patients ranging from approximately 20% to 40%.

## Conclusions

Most (71%) women in this laparoscopic hysterectomy cohort did not receive any narcotics. This is likely attributable to the aggressive use of non-narcotic analgesics. There was unexplained between-surgeon variability in patient narcotic usage. Routine non-prn (non-discretionary) order sets offer an attractive therapeutic option for the management of post-op pain.

